# Interrupted sutures prevent recurrent abdominal fascial dehiscence: a comparative retrospective single center cohort analysis of risk factors of burst abdomen and its recurrence as well as surgical repair techniques

**DOI:** 10.1186/s12893-021-01219-x

**Published:** 2021-04-26

**Authors:** Matthias Mehdorn, Linda Groos, Woubet Kassahun, Boris Jansen-Winkeln, Ines Gockel, Yusef Moulla

**Affiliations:** Department of Visceral, Transplant, Thoracic and Vascular Surgery, University Hospital of Leipzig, Liebigstraße 20, 04103 Leipzig, Germany

**Keywords:** Burst abdomen, Abdominal fascial dehiscence, Burst abdomen recurrence, Interrupted sutures, IPOM augmentation

## Abstract

**Background:**

Burst abdomen (BA) is a severe complication after abdominal surgery, which often requires urgent repair. However, evidence on surgical techniques to prevent burst abdomen recurrence (BAR) is scarce.

**Methods:**

We conducted a retrospective analysis of patients with BA comparing them to patients with superficial surgical site infections from the years 2015 to 2018. The data was retrieved from the institutional wound register. We analyzed risk factors for BA occurrence as well as its recurrence after BA repair and surgical closure techniques that would best prevent BAR.

**Results:**

We included 504 patients in the analysis, 111 of those suffered from BA. We found intestinal resection (OR 172.510; 22.195–1340.796, p < 0.001), liver cirrhosis (OR 4.788; 2.034–11.269, p < 0.001) and emergency surgery (OR 1.658; 1.050–2.617; p = 0.03) as well as postoperative delirium (OR 5.058; 1.349–18.965, p = 0.016) as the main predictor for developing BA. The main reason for BA was superficial surgical site infection (40.7%). 110 patients received operative revision of the abdominal fascial dehiscence and 108 were eligible for BAR analysis with 14 cases of BAR. Again, post-operative delirium was the patient-related predictor for BAR (OR 13.73; 95% CI 1.812–104-023, p = 0.011). The surgical technique of using interrupted sutures opposed to continuous sutures showed a preventive effect on BAR (OR 0.143, 95% CI 0.026–0,784, p = 0.025). The implantation of an absorbable IPOM mesh did not reduce BAR, but it did reduce the necessity of BAR revision significantly.

**Conclusion:**

The use of interrupted sutures together with the implantation of an intraabdominal mesh in burst abdomen repair helps to reduce BAR and the need for additional revision surgeries.

## Background

Burst abdomen (BA), or abdominal fascial dehiscence, is a serious complication in abdominal surgery, with an incidence of about 1% [1, 2] in elective surgeries and an even higher frequency after emergency surgery [3]. The occurrence of fascial dehiscence represents a risk factor for increased mortality rates of up to 25% [4, 5]. It is characterized by a breakdown of all abdominal layers [6, 7]. In the early postoperative phase with non-adherent bowel urgent repair of the abdominal wall is mandatory to prevent evisceration and infection of the abdominal cavity [6, 7] as a delayed closure might increase morbidity [6]. Only small fascial defects, adherent bowel or poor patient conditions might favor a conservative approach [7].

Risk factors for abdominal fascial dehiscence have been studied in the past. As most important risk factors, surgical site infections [4, 5, 8–10], coughing or chronic lung disease [5, 8, 10] and hypoalbuminemia have been mentioned [9, 10]. Other risk factors have been suggested but were significantly predominant in certain cohorts, i.e. chronic steroid use [4, 5], diabetes mellitus [9] or hypertension [5]. Combining several common risk factors has been used to establish risk stratification scores for the preoperative risk assessment of BA development in the US [11] and the Netherlands [5]. Diagnostic accuracy has been validated to be acceptable [4] (sensitivity and specificity of 94% and 48% [11] and 98% and 20% [5], respectively).

There have been multiple studies on the best modalities of fascial closure after elective abdominal surgery procedures, comparing continuous to interrupted or absorbable to non-absorbable sutures. In the end, no significant differences have been found with regard to burst abdomen or incisional hernia in meta-analyses [12, 13]. Generally, the risk of incisional hernia after abdominal surgery ranges from 12 to 25% [1, 14] and reaches incidences as high as 83% after burst abdomen [15].

So far, previous studies and existing data mainly focus on primary abdominal closure after surgery with the aforementioned outcome parameters. Nevertheless, evidence is scarce on how to close burst abdomen to prevent its recurrence. Past studies have raised evidence that retention sutures should not be used in abdominal fascial dehiscence repair [16, 17]. Recently, Jensen et al. stated that the introduction of a small-bites technique with continuous slowly absorbable suture reduced the recurrence of burst abdomen remarkably [18]. This reflects the current recommendations of the European Hernia society (EHS) to use slowly absorbable monofilamentous continuous sutures in burst abdomen repair. Those recommendations are, nonetheless, based on clinical expertise but not on study data [19].

Although, implanting a prosthetic, non-absorbable mesh during burst abdomen repair surgery seemed to prevent recurrence of fascial dehiscence and did reduce incisional hernia [20–22], significant morbidity due to mesh infections has been reported. It appears that even the use of absorbable meshes results in relevant mesh related complications [23].

Thus, the question remains, which strategy might be the best to avoid recurrence of burst abdomen in accordance with its respective etiology, taking into account suture techniques and techniques for abdominal wall reinforcement.

We therefore conducted an analysis of our prospective clinical wound register, with the aim to find out the risk factors for BA and its recurrence and if a certain suturing technique or the implantation of meshes would prevent burst abdomen recurrence (BAR).

## Methods

### Patient inclusion and stratification

We implemented an institutional wound register in our hospital, a tertiary referral hospital, for abdominal superficial surgical site infections (SSSIs) in 2019. In this study, we report the results for the time span from 2015 to 2018 of our retrospective cohort.

For patient inclusion, we sought the electronic patient charts for ICD-codes of infectious complications (T81.3, T81.4, T81.8) and OPS codes for wound debridement or V.A.C. therapy. We included all abdominal procedures of general and abdominal surgery. Patients with abdominal vascular procedures, i.e. abdominal aortic repair, were excluded due to the low case-load of open opposed to endovascular procedures in our institution. Furthermore, abdominal closure in these operations was not performed by abdominal surgeons. We also excluded patients with organ space abscesses with no need for surgical intervention, if this was their only SSI. Patients who were referred to our department after primary surgery elsewhere, were excluded to ensure data quality. Follow-up is based on the electronic patient charts.

**Fig. 1:**
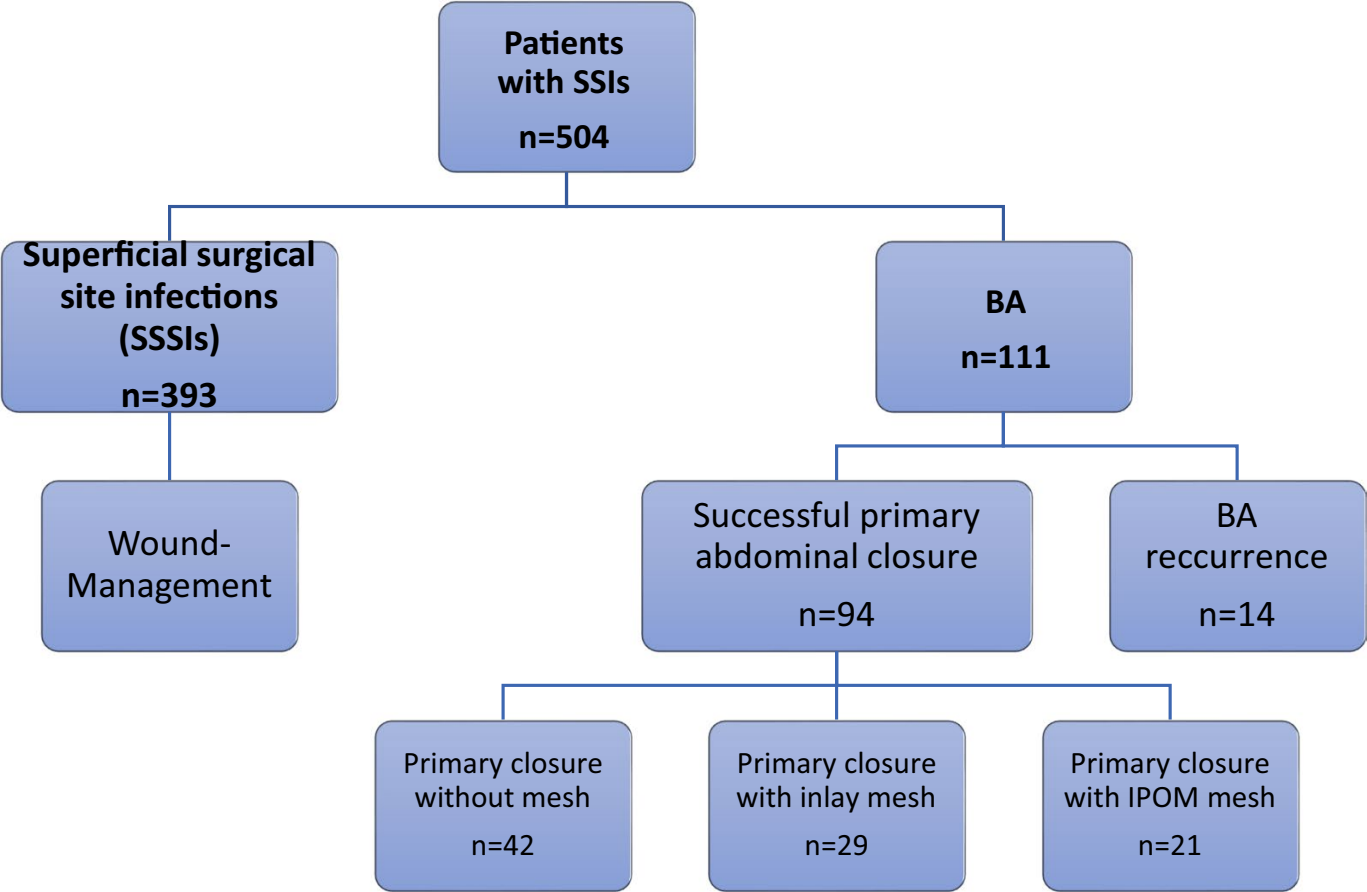
Algorithm of included patients and subgroups. *SSI* surgical site infections; *BA* burst abdomen; Of 111 patients one received a conservative treatment, two were excluded from further analysis as they were operated on for other causes than recurrent BA

The analyzed groups and subgroups are presented in Fig. 1.

### Surgical site infections (SSI)

According to the definition of the American “Center for Disease Control” (CDC), a postoperative superficial surgical site infection (SSSI) is when: (1) the surgical wound shows signs of local inflammation (swelling, tenderness, purulent secretion, erythema, pain), (2) the treating physician opens the wound or (3) a pathogen can be isolated from the wound within 30 days from surgery. For the definition of burst abdomen, we refer to the definition given in the introductory paragraph.

In our department, we have a specialized group of nurses, who are dedicated to wound care of complex wounds. All wounds are documented in the electronic patient chart with the respective measures and the applied dressings.

We administer cefuroxime and metronidazole as single shot antibiotics preoperatively or cefuroxime only in cases of upper GI surgery for SSI prevention.

### Surgical technique

In our department, all median laparotomies are closed with 2 looped number 1 PDS sutures (Ethicon, Johnson&Johnson, Norderstedt, Germany) in a mass suturing technique. Oblique incisions are closed with two separate running PDS sutures for anterior and posterior rectus sheath. BA is diagnosed clinically during wound inspection on the surgical ward or occasionally during revision laparotomy. Generally, a thorough exploration of the abdominal cavity is performed during revision surgery to check for intra-abdominal pathologies or other underlying causes for BA. Depending on the fascial quality, the fascial defect will be closed either with continuous looped number 1 PDS or interrupted number 2 Vicryl sutures (Ethicon, Johnson&Johnson, Norderstedt, Germany) according to the surgeon’s discretion. If the fascial quality seems insufficient for primary fascial closure or would be closed with tension, a Vicryl mesh can be placed in IPOM position before the fascia is closed. If closure of the fascial defect is impossible due to extensive fascial necrosis or intestinal edema, a Vicryl mesh will be placed in the inlay position to prevent evisceration and, subsequently, a V.A.C. dressing (KCI, Acelity, Wiesbaden, Germany) will be installed to create a laparostomy.

### Clinical parameters

Of all patients, the following patient inherent and procedure specific parameters were assembled in our database: primary procedure, main diagnosis, comorbidities and data of their postoperative course, including general complications and length of stay (LOS). Additionally, we recorded data about the operative setting, such as emergent or elective procedure, type of laparotomy, surgical approach (laparoscopic or open), duration of primary surgery (DOS), wound contamination class according to CDC and postoperative ICU stay. Albumin levels at the time of SSI development were recorded if available.

We collected further information for the patients who developed a BA: condition of abdominal fascia as reason for BA, total number of revisions, type of BA closure, implantation of mesh and its respective position within the abdominal wall (intraperitoneal onlay mesh = IPOM, fascial bridging = inlay), recurrence of BA, postoperative enteroatmospheric fistula and if skin closure was possible during the course of treatment. The data on reason for BA and technical features of BA closure as well as the presence of intraabdominal abscesses were taken from the operation report of the BA closure procedure. The reason for BA is classified as technical with unimpaired fascial quality (i.e. breaking of suture material), superficial infection (if a subcutaneous wound infection existed with simultaneous fascial dehiscence) or fascial necrosis (if fascia mainly was necrotic with a vital non-infected subcuticular layer).

### Statistical analysis

We calculated frequencies of all dichotomous variables. For continuous variables we tested for normal distribution and calculated means with standard deviation (± SD) or median with interquartile range (IQR).

In the univariate analysis, the unpaired t-test or Mann–Whitney-U-test was applied when testing for variance of continuous variables as well as the Chi^2^-Test or exact Fisher Test for dichotomous or categorical variables. Significance levels were set at p = 0.05.

We used the binary logistic regression model with backward stepwise selection to test for specific risk factors of BA. We included all factors from the univariate analysis that had reached a p-value of ≤ 0.1 and considered results statistically significant in the multivariate analysis if the p-value was ≤ 0.05. All risk factors are expressed as odds ratio (OR) with their respective 95% confidence interval (95% CI).

For data acquisition, we used Excel 2016 (Microsoft, München, Germany) and SPSS 25 for data analysis (IBM statistics, Ehningen, Germany).

The study is reported adhering to the STROCCSS 2019 statement on reporting of cohort studies in surgery [24].

## Results

### Patients’ characteristics

In total, 504 patients with abdominal surgical site infections were enrolled in the study between 2015 and 2018. There were 207 (41.1%) females and 297 (58.9%) males. Of those, a total of 111 patients had BA, of which 69.4% were male, compared to 56% males in the superficial surgical site infections group (SSSI, p = 0.011). The demographic and procedure specific data of the whole cohort and the respective subgroups (BA group and SSSI group) are shown in Tables 1 and 2, whereas Table 3 displays the relevant postoperative complications of those groups.

**Table 1:** Patient comorbidities

	All patients (n = 504)	Burst abdomen (n = 111)	Superficial SSI other than burst abdomen (n = 393)	p value
*Sex: male/female (%)*	58.9 /41.1	69.4/30.6	56/44	0.011*
*Age in years*	62.18 (± 14.76)	63.59 (± 14.58)	61.786 (± 14.811)	0.254
*Patient comorbidities (%)*				
Hypertension	62.1	64.9	61.3	0.497
Peripheral artery disease	3.8	3.6	3.8	0.917
Congestive heart failure/coronary artery disease	24.6	26.1	24.2	0.673
Liver cirrhosis	11.7	18.7	9.7	0.007*
Terminal renal insufficiency	5.4	6.3	5.1	0.619
Diabetes mellitus	25.4	20.7	26.7	0.200
Dementia	1.2	1.8	1.0	0.501
Past or present malignant disease	58.3	57.7	58.8	0.870
Past or present chemotherapy	24.4	24.3	24.4	0.982
Immunosuppressants	18.3	21.6	17.3	0.298
Chronic inflammatory disease	11.3	16.2	9.9	0.065
BMI in kg/m^2^	25.75 (IQR 48.3)	26.35 (IQR 7.6)	25.7 (IQR 6.5)	0.542

All values are given as percentage or median (interquartile range)

*Significance level was set at p < 0.05

*BMI* body mass index

**Table 2:** Procedure specific data

	All patients (n = 504)	Burst abdomen (n = 111)	Superficial SSI other than burst abdomen (n = 393)	p value
*Most frequent pathologies (*> *5%)(%)*				
Malignancy lower GI	16.7	11.7	18.1	
Malignancy liver	13.3	18.9	11.7	
Lower GI inflammation	12.7	14.4	12.2	
Ileus	8.3	8.1	8.4	
Abdominal wall hernia	8.3	3.6	9.7	
Liver transplantation	7.3	9	6.9	
Malignancy pancreas	6	5.4	6.1	
Malignancy upper GI	5.2	5.4	5.1	
Mesenteric ischemia	1.6	5.4	0.5	
*Surgical approach (%)*				
Open/Laparoscopic	93.3/6.7	94.6/5.4	92.9/7.1	0.524
*Most common types of laparotomy (%)*				
Median laparotomy	50	53.2	49.1	
Transverse abdominal incision	12.3	13.5	12.0	
Transverse abdominal incision with median epigastric incision	13.9	20.7	12.0	
Median laparotomy with subcostal incision	5.4	3.6	5.9	
Pfannenstiel's incision	2.6	3.6	2.3	
*Wound contamination class (%)*				
Clean	7.1	6.3	7.4	0.713
Clean-contaminated	46.6	46.8	46.3	
Contaminated	19.2	22.5	18.3	
Dirty	27.2	24.3	28.0	
*Duration of surgery in min*	244.50 (± 144.109)	248.70 (± 148.715)	242.32 (± 142.32)	0.745
*Intestinal resection (%)*	22.6	42.3	17.0	< 0.001*
*Emergency surgery (%)*	36.5	49.5	32.8	0.001*

All values are given as percentage or mean (± standard deviation)

*Significance level was set at p < 0.05

*SSI* surgical site infection, *GI* gastrointestinal tract, *BA* burst abdomen

**Table 3:** Post-operative course and complications

	All patients (n = 504)	Burst abdomen (n = 111)	SSI other than burst abdomen (n = 393)	p value
ICU stay (%)	75.4	83.8	73	0.022*
Length of ICU stay in days	3 (IQR 70)	4.00 (IQR 9)	3.00 (IQR 5)	0.131
Total length of stay in days	28 (IQR 20)	32.50 (IQR 18)	26.00 (IQR 22)	< 0.001*
*Postoperative complications (%)*				
Cardioembolic	8.7	13.5	7.4	0.043*
Pneumonia	7.5	9.0	7.1	0.507
Delirium	5.8	10.8	4.3	0.010*
Bleeding	6.2	11.7	4.6	0.058
Acute renal failure	35.5	45.9	32.6	0.010*
Death	4.8	9.9	3.3	0.004*
Albumin at time of SSI	27.39 (± 5.96)	28.01 (± 5.97)	25.30 (± 5.46)	0.002*

All values are given as percentage or mean (± standard deviation)

*Significance level was set at p < 0.05

*V.A.C.* vacuum assisted closure

All in all, patients with abdominal fascial dehiscence stayed approximately 6.5 days longer in hospital compared to the SSSI group (32.50 days (IQR 18) and 26.00 days (IQR 22); p < 0.001).

### Univariate analysis of risk factors for burst abdomen (BA)

There was no difference between BA group and SSSI group with regard to wound contamination class (p = 0.713), duration of surgery (0.745), and surgical approach (p = 0.524). But most of the SSI occurred after open surgery (> 90%).

Sex, intestinal resections, emergency setting and liver cirrhosis reached the significance level in the univariate analysis und have been entered into multivariate analysis (Tables 1 and 2).

Chronic inflammatory disease as risk factor did not reach significance levels (p = 0.065) but will be considered for further analysis according to our criteria mentioned above.

Patients with burst abdomen suffered more often from acute renal failure (p = 0.01), delirium (p = 0.01) and had higher mortality rates (9.9% vs 3.3%; p = 0.004). Additionally, the plasma albumin levels at time of SSI were lower in the BA group compared to the SSSI group (p = 0.002).

### Multivariate analysis of risk factors for BA

The multivariate analysis revealed the following parameters as significant risk factors for BA: Intestinal resection (OR 4.006; 2.456–6.535, p < 0.001), liver cirrhosis (OR 2.568; 1.355–4.866, p = 0.004) and emergency surgery (OR 1.658; 1.050–2.617, p = 0.03). Sex turned out to be an important risk factor as well but failed to reach significance levels (p = 0.08).

Adding the postoperative complications to the regression model (acute renal failure, delirium and bleeding) eliminated emergency surgery from the regression model and included delirium (OR 5.058; 1.349–18.965, p = 0.016). Furthermore, in this newly calculated regression model, the odds ratios of the previously considered risk factors for the development of an abdominal fascial dehiscence also increased, especially for intestinal resection (OR 172.510; 22.195–1340.796, p < 0.001) and to a lesser extent for liver cirrhosis (OR 4.788; 2.034–11.269, p < 0.001).

Albumin levels at time of SSI were a significant risk factor (OR 0.911; 0.861–0.986, p = 0.001). As this parameter was only available in 270 patients, we disregarded it in the final analysis, although it hints at low albumin levels as risk factor for BA.

### Characteristics of patients with burst abdomen (BA group)

Of all burst abdomen patients (n = 111), 110 (99.1%) had an operative revision. One patient (0.9%) was treated conservatively, which was due to a palliative situation and a limited fascial dehiscence.

Abdominal fascial dehiscence was mostly caused by superficial infections (40.7%), fascial necrosis (20.4%) or technical issues (13.9%). An intrabdominal abscess was present in 12.7% of the revision laparotomies.

Technical details of BA closure can be found in Table 4. During BA repair, complete fascial closure was achieved in 69.4%, in the rest of the cases no fascial closure was possible and subsequently an absorbable mesh was implanted in inlay position for fascial bridging. No significant tendency could be found as preferred method of fascial closure (type of suture with or without IPOM mesh, p = 0.381).

**Table 4:** Technical aspects of burst abdomen und its closure

	Overall burst abdomen group (n = 108)	BA revision group (n = 94)	Recurrent BA group (n = 14)	p value
*Fascial condition/reason for BA* *(absolute number/relative frequency)*				
Unimpaired fascia/technical	15 (13.9)	13 (13.8)	2 (14.3)	0.47
Superficial SSI	44 (40.7)	40 (42.6)	4 (28.6)	
Fascial necrosis	22 (20.4)	17 (18.1)	5 (35.7)	
Not indicated	27 (25)	24 (25.5)	3 (21.4)	
*Surgical techniques in patients with complete fascial closure at BA revision*				
Suture type and technique (n = 69)				
PDS continuous	25 (36.2)	18 (31)	7 (63.6)	0.039*
Vicryl interrupted	44 (63.8)	40 (69)	4 (36.4)	
Vicryl mesh augmentation (n = 75)				
No mesh	51 (68)	43 (67.2)	8 (72.7)	0.716
IPOM mesh	24 (32)	21 (32.8)	3 (27.3)	

All values are given as absolute numbers (relative frequencies). Fascial condition was lacking in two patients. The data for the type and technique of suture was lacking in six patients

*Significance level was set at p < 0.05

*BA* burst abdomen, *SSI* surgical site infection, *PDS* polydioxanone, *IPOM* intraperitoneal onlay mesh

Two patients developed enteroatmospheric fistula, one after IPOM mesh implantation, the other after complete fascial closure.

### Univariate analysis of risk factors of burst abdomen recurrence (BAR)

For the evaluation of burst abdomen recurrence (BAR), two additional patients had to be excluded from the analysis as they were reoperated on for other reasons than recurrent abdominal fascial dehiscence.

The patient comorbidities and post-operative complications for the comparison of BA and BAR groups can be found in Tables 4 and 5. A recurrence of abdominal fascial dehiscence took place in 14 cases (13%). From that data, no significant differences between those two groups can be deduced with regards to comorbidities or surgical techniques. The only significant difference was the type and technique of suture that was used for BA closure (p = 0.039). Interrupted Vicryl sutures were more commonly used in the BA group without recurrence.

**Table 5:** Risk factors for recurrent BA

	BA revision group (n = 94)	Recurrent BA group (n = 14)	p value
Sex: male /female (%)	65 /29	10 /4	0.863
Age (mean ± SD)	63.227 (± 14.487)	64.294 (± 16.403)	0.785
*Surgery related factors* *(absolute number/relative frequency)*			
Intestinal resection	41 (43.6)	3 (21.4)	0.115
Emergency	48 (51.1)	4 (28.6)	0.116
*Comorbidities* *(absolute number/relative frequency)*			
Hypertension	57 (60.6)	12 (85.7)	0.068
Peripheral artery disease	3 (3.2)	1 (7.1)	0.465
Congestive heart failure/coronary artery disease	21 (22.3)	6 (42.9)	0.098
Liver cirrhosis	19 (20.2)	2 (14.3)	0.601
Diabetes mellitus	18 (19.1)	5 (35.7)	0.158
Dementia	1 (1.1)	1 (7.1)	0.115
Past or present malignant disease	53 (56.4)	19 (71.4)	0.287
Past or present chemotherapy	23 (24.5)	4 (28.6)	0.741
Immunosuppressants	22 (23.4)	2 (14.3)	0.444
Chronic inflammatory disease	16 (17)	2 (14.3)	0.798
BMI in kg/m^2^ (median + IQR)	26.2 (6.9)	30.2 (11.3)	0.537
*Postoperative complications* *(absolute number/relative frequency)*			
Cardioembolic event	11 (11.7)	2 (14.3)	0.782
Pneumonia	9 (9.6)	1 (7.1)	0.770
Acute renal failure	41 (43.6)	8 (57.1)	0.343
Delirium	8 (8.5)	4 (28.6)	0.026*
Bleeding	10 (17.2)	2 (40)	0.233
Intraabdominal abscess	11 (11.7)	2 (14.3)	0.782

All values are given as absolute numbers (relative frequency) or median (interquartile range)

*Significance level was set at p < 0.05

Although the implantation of an IPOM mesh did not reduce BAR (p = 0.678), the patients who had received an IPOM mesh during revision laparotomy, needed significantly fewer operative revisions for BAR (0/3 = 0% versus 8/8 = 100%, p = 0.001).

### Multivariate analysis of risk factors for BAR

All risk factors with a p ≤ 0.1 in the univariate analysis were included in the stepwise binary logistic regression modell: CAD/CHF, hypertension, type of suture, postoperative delirium. Finally, postoperative delirium was the strongest predictor for developing BAR (OR 13.73; 95% CI 1.812–104-023, p = 0.011). Whereas, using interrupted Vicryl stitches as fascial closure technique confirmed to be a preventive factor of BAR in the multivariate analysis (OR 0.143, 95% CI 0.026–0,784, p = 0.025).

## Discussion

In this analysis of our institutional wound register, we evaluated the risk factors for BA and RBA and the best surgical treatment options of burst abdomen after abdominal operations with an emphasis on burst abdomen recurrence.

The overall number of patients included in this study is among the largest studies in this field [18, 20, 22, 25]. First and foremost, the reference group in our study is not a standard control group of healthy individuals with uneventful postoperative recoveries, but patients suffering from postoperative superficial surgical site infections of all kinds. Therefore, it represents a selected collective which may over-represent risk factors for infectious complications or burst abdomen rates. Still, the burst abdomen patients showed several specific characteristics compared to the control group.

### Risk factors for BA

Of all comorbidities and procedure-specific parameters, liver cirrhosis was an important risk factor for BA, which has not been reported by other studies that have evaluated scores for the occurrence of BA [4]. So far, Ramshorst et al. found an impaired liver function (jaundice, ascites) to be associated with burst abdomen [5]. In that study, even less severe comorbidities, such as hypertension, were significantly increased in the BA group, which is contradictory to our findings. That variance may be due to the aforementioned preselection in our register. Tolstrup et al. described liver cirrhosis in emergency surgeries as a main risk factor for the development of burst abdomen [3].

Furthermore, emergency surgery was a risk factor for BA in our analysis as well as in literature [3, 18]. But the most important risk factor for the development of abdominal wall dehiscence was, nonetheless, intestinal resection with an OR of 4 in the regression analysis of preoperative risk factors and an OR of 172 in the combined regression model of preoperative and postoperative risk factors including liver cirrhosis and emergency surgery. An accidental contamination of the surgical wound with intestinal flora might explain the high rate of SSI and BA in that group. With that in mind, preoperative selective intestinal decontamination might be immensely important to reduce harmful intestinal bacterial flora. Though, such an approach would not have been possible for the 30% of patients who underwent emergency surgery.

Additionally, according to preoperative and postoperative findings, delirium was an important predictor for BA. To our knowledge, no other reports mention delirium as a risk factor. Nevertheless, it seems plausible, on one hand, that a patient with an active delirium, marked by fierce agitation, would not adhere to nursing instructions and applies strong forces to his abdominal wall putting it at risk for dehiscence. Or, on the other hand, that a patient with a prolonged stay, due to burst abdomen, would be more vulnerable to developing a delirium. The same thoughts and reasons might apply to the development of RBA. So, if a patient with a prolonged stay and an additional surgical procedure for BA develops a delirium the already weakened abdominal wall (i.e. necrotic fascial layers) does not resist agitation forces and thus is prone to reburst.

In conclusion, patients with liver cirrhosis who underwent emergency surgery that included intestinal resection and who developed a post-operative delirium had the highest risk of BA development.

The patients in our BA group had higher mortality rates compared to the SSI group. Although, regression analysis did not prove BA to be an independent risk factor for mortality, as neither of the complications did. As the BA group had more overall complications, a longer ICU stay and a longer in-hospital stay, mortality rates are a surrogate variable for that complication prone group of patients. But overall, our mortality rate of 9.9% is comparably low to other studies that showed mortality rates of up to 35% in patients with BA [25].

### Intraoperative findings during BA revision surgery

Only few studies have analyzed causes of BA during revision surgery for abdominal wall dehiscence. Graham reported that intraabdominal abscesses were diagnosed as primary reason for BA in about 50% of the revision laparotomies for BA and thus advocated for aggressive decision making towards revision laparotomies instead of conservative treatment of BA [25]. However, we found intraabdominal abscesses in only 12% of the revision laparotomies for BA repair. This lower number might be due to the availability of interventional radiology for abscess drainage nowadays so that a solitary intraabdominal abscess rather would not cause an abdominal fascial dehiscence.

We classified the underlying cause of BA according to fascial quality in three groups: superficial infection (40.7%), fascial necrosis (20.4%) and technical with unimpaired fascia (13.9%). Mäkelä et al. and Ramshorst et al. identified the cutting out of sutures through the tissues as main cause for BA [5, 10]. The major problem might be the exact definition of “cutting of the sutures through the tissues” [10] as that same phenomenon occurs in fascial necrosis or in tissue lysis due to superficial infection. Overall, in our cohort, only the smaller part of the BAs occurred when the fascia was unaffected, i.e. technical failure. Moreover, our classification of the BA causes is based on tissue quality but does not exclude the surgeon or surgical technique of primary fascial closure as reason for abdominal wall break down. There is evidence that the necessity of multiple laparotomies increases the risk of burst abdomen [26], maybe another factor for decreased fascial tissue stability. Our data does not support those findings, as for most patients, BA repair was the first revision laparotomy. Furthermore, decreased plasma albumin levels were a risk factor for BA development compared to patients with superficial SSI. Hence, decreased protein plasma levels also lead to an impaired wound healing and decreased fascial stability.

### Suture techniques for BA repair

The findings of impaired fascial tissue quality as main reason for BA lead to the central question of what would be the best closure technique for BA repair to prevent repeated fascial dehiscence.

A recurrence of BA occurred in 12.9%, which is in the middle of the reported frequencies of RBA [15, 18, 25, 27]. The vast difference in RBA frequency is probably due to cohort selection bias, i.e. emergency surgery compared to elective surgery. Jensen et al. recently stated that the use of small bites PDS running sutures would reduce recurrent abdominal wound dehiscence by the factor of 3 [18]. In contrast to their findings, our study points out the running suture with PDS as the major surgical risk factor of BAR with a superiority of interrupted Vicryl sutures. Our BA recurrences were diagnosed within fourteen days from the initial BA repair. Therefore, we would attribute the difference in BAR not to the suture material itself but the technique of continuous versus interrupted stitches, as both suture materials obtain similar tensile strength in that period. The BAR group had a slightly higher frequency of fascial necrosis and was more often closed with continuous sutures. Hence, we conclude, that a continuous suture should not be used in case of poor fascial quality. Lopez-Cano et al. recommended the use of continuous monofilamentous sutures (i.e. PDS) in their European Hernia Society (EHS) guidelines for the closure of BA [19], with the main focus on incisional hernias but not on BAR or SSI. They also pointed out the lack of appropriate data to support their recommendation. Underlined by our data, we cannot support that recommendation with regard to BAR. Instead, we suggest the closure of abdominal fascial dehiscence with interrupted sutures to best prevent its recurrence, as its main cause seems to be necrosis and destruction of the fascial layers.

The incidence of incisional hernias that can be attributed to a certain suture type has been subject to fierce discussion in the surgical literature and was summed up by a recently published Cochrane review. The authors could not draw an evidence-based conclusion with regard to the most suitable suture materials which prevents all adverse events most effectively [13]. In our cohort, we currently do not provide any data on incisional hernias but further follow-up will clarify that issue.

### Mesh implantation in BA repair

The implantation of meshes during primary BA repair has been reported to reduce incisional hernia incidence and even burst abdomen recurrence [22]. In regards to mesh implantation, current knowledge advocates the use of a non-absorbable mesh during definitive BA repair to prevent further incisional hernias. Nevertheless, a significant rate of SSI has been reported by multiple studies after the use of non-absorbable meshes [19–21], and even after the implantation of absorbable meshes [23]. The exact position of the mesh (i.e. IPOM or sublay) was not specified in most studies. We found no significant benefit of IPOM Vicryl mesh implantation to reduce the incidence of recurrent BA, but our data clearly shows a reduced revision rate for recurrent burst abdomen (0% versus 100% revision rate), because the mesh prevents evisceration. This could save the patient an additional surgical procedure without significantly increasing the risk for incisional hernias as it is up to 83% for burst abdomen patients anyways [15]. This might be especially important in critically ill patients who would suffer a severe setback by repetitive surgical procedures. None of our patients had to be reoperated on due to mesh related complications (migration, stricture, sinus). Yet, one of them developed an enterocutaneous fistula. On the other hand, one patient without a mesh also showed fistula formation. Similar findings have been reported before [22], so we consider the implantation of a Vicryl mesh in IPOM position safe and feasible and recommend it to reduce the need of revision laparotomy in case of BAR.

## Study limitations

Our study has several limitations that are mostly based on its retrospective nature. We tried to obtain the best data quality possible by only including patients who were primarily treated in our department so that complete data sets were available. Data quality was also assured by the continuity of our wound care specialists over the whole period of the study. Our cohort is within the range of the largest burst abdomen studies published and, although the subgroup analyses rely on comparably large numbers, the absolute number of patients per subgroup remains limited. Thus, completing the retrospective cohort with a relevant number of patients from the prospective cohort will provide extra information. Further research could focus on prospective comparison of different suture types with regard to BAR.

Further follow-up of our cohort will clarify the relation between suture techniques, BAR and incisional hernia.

## Conclusion

We could add further evidence to the risk factors of developing a BA by stressing the importance of intestinal resection, liver cirrhosis and emergency surgery as well es post-operative delirium. Furthermore, we found delirium to be the only patient related predictor for recurrent BA. With regard to surgical technique, we could show the superiority of interrupted sutures in BA repair to best prevent its recurrence when considering BA etiology. The additional implantation of an absorbable mesh in IPOM position reduces operative revision rates after BAR.

In our opinion, abdominal closure with interrupted sutures and implantation of an absorbable mesh in IPOM position is recommended in patients with BA.

## Data Availability

The raw data of this study is accessible from the corresponding author on reasonable request. It has not been stored in a publicly available data repository. For access of the electronic patient chart the ethics review board granted access to those data for the purpose of this specific study. The data of the institutional wound register are under supervision und responsibility of the first author who has been approved as principal investigator by the ethics review board.

## Abbreviations

BA: Burst abdomen
BAR: Burst abdomen recurrence
CDC: Center for Disease Control and Prevention
DOS: Duration of surgery
EHS: European Hernia Society
ICD: International Classification of Diseases
IPOM: Intraperitoneal Onlay Mesh
IQR: Inter quartile range
LOS: Length of stay
OPS-Code: German Operation and Procedure Coding system
OR: Odds ratio
PDS: Polydioxanone suture
SD: Standard deviation
SSI: Surgical site infection
SSSI: Superficial surgical site infection
V.A.C.: Vacuum assisted closure
95%CI: 95% Confidence interval

## Declarations

### Study registration

The study was retrospectively registered in the German register for clinical trials (DRKS, DRKS00019058, 19th December 2019; https://www.drks.de/drks_web/navigate.do?navigationId=trial.HTML&TRIAL_ID=DRKS00019058).
